# Updates in Gastrointestinal Oncology – insights from the 2008 44^th ^annual meeting of the American Society of Clinical Oncology

**DOI:** 10.1186/1756-8722-2-9

**Published:** 2009-02-23

**Authors:** Milind Javle, Chung-Tsen Hsueh

**Affiliations:** 1 Department of Gastrointestinal Medical Oncology, The University of Texas MD Anderson Cancer Center, Houston, TX 77030, USA; 2 Division of Medical Oncology and Hematology, Loma Linda University, Loma Linda, CA 92354, USA

## Abstract

We have reviewed the pivotal presentations rcelated to colorectal cancer (CRC) and other gastrointestinal malignancies from 2008 annual meeting of the American Society of Clinical Oncology (ASCO). We have discussed the scientific findings and the impact on practice guidelines and ongoing clinical trials. The report on KRAS status in patients with metastatic CRC receiving epidermal growth factor receptor (EGFR) targeted antibody treatment has led to a change in National Comprehensive Cancer Network guideline that recommends only patients with wild-type KRAS tumor should receive this treatment. The results of double biologics (bevacizumab and anti-EGFR antibody) plus chemotherapy as first-line treatment in patients with metastatic CRC has shown a worse outcome than bevacizumab-based regimen. Microsatellite Instability has again been confirmed to be an important predictor in patients with stage II colon cancer receiving adjuvant treatment.

Adjuvant gemcitabine therapy for pancreatic cancer was investigated by the CONKO-001 study; this resulted in superior survival as compared with observation and can be regarded as an acceptable option, without the addition of radiotherapy. The addition of bevacizumab to gemcitabine and erlotinib was not supior to gemcitabine and erlotinib for advanced disease. Second-line therapy for advanced pancreatic cancer with 5-fluorouracil and oxaliplatin resulted in a survival benefit. Irinotecan plus cisplatin and paclitaxel plus cisplatin result in similar survival when combined with radiotherapy for esophageal cancer. The novel fluoropyrimidine S1 appears to be active in gastric cancer, as a single agent or as combination therapy. Adjuvant intraperitoneal mitomycin-C may decrease the incidence of peritoneal recurrence of gastric cancer. Sorafenib is an effective agent in Asian patients with hepatocellular carcinoma secondary to hepatitis B; its utility in child's B cirrhosis remains to be proven. Sunitinib is also an active agent in hepatocellular carcinoma, and may represent an alterative to sorafenib for advanced disease. These and other important presentations from the 2008 ASCO annual meeting are discussed in this article.

## Colorectal cancer

Colorectal cancer (CRC) is among the top three most common malignancies and cancer-related death in Western world including United States [1]. In 2008, it is estimated about 150,000 new cases, and approximate 50,000 patients die from this disease. The mortality for this disease has decreased slightly over the past three decades, mainly due to improvement in screening and treatment. For patients with early stage disease, surgery is the main treatment, and frequently patients will benefit from adjuvant treatment. The selected presentations from 2008 annual meeting of American Society of Clinical Oncology (ASCO) are grouped into three categories: metastatic CRC, adjuvant chemotherapy in stage II/III colon cancer, and neurotoxicity and efficacy with intermittent oxaliplatin and use of calcium and magnesium.

## Metastatic colorectal cancer

### KRAS Mutation Predicts Lack of Response to Epidermal Growth Factor Receptor Antibody Treatment

Cetuximab and panitumumab are epidermal growth factor receptor (EGFR) targeted antibodies approved for clinical use in patients with metastatic CRC. Ligand binding of the EGFR activates the RAS/RAF/MAPK, STAT, and PI3K/AKT signaling pathways, which modulate cellular proliferation, angiogenesis, and survival. However, the level of EGFR expression as measured by immunohistochemistry does not predict clinical benefit [2].

KRAS, the human homolog of the Kirsten rat sarcoma-2 virus oncogene, encodes a small GTP-binding protein, and acts as signal transducer in response to ligand binding of growth factor receptor, including EGFR [3]. KRAS can harbor oncogenic mutation, mostly in codon 12 and 13, that yields a constitutively active protein, and such mutation is found in approximately 30% to 50% of CRC [4]. Several retrospective analyses of tumor samples in CRC patients receiving anti-EGFR antibody treatment have shown that patients with mutated KRAS did not benefit from anti-EGFR therapy [5,6]. Three clinical studies analyzing KRAS status retrospectively in metastatic CRC patients have further supported this finding.

The CRYSTAL study is a phase III study comparing first-line chemotherapy with a regimen of 5-fluorouracil (5-FU), leucovorin (LV) and irinotecan, known as FOLFIRI, with or without cetuximab. At 2007 ASCO annual meeting, data from the CRYSTAL study was first presented, which showed that addition of cetuximab to FOLFIRI increased response rate (RR) by 8% and prolonged progression-free survival (PFS) by 0.9 months [7]. At plenary session of 2008 ASCO annual meeting, Dr. Eric Van Cutsem presented a retrospective analysis of KRAS data in archived tumor tissues obtained from 540 of the 1,198 patients enrolled in CRYSTAL study [8]. Mutated KRAS was detected in 192 patients (36%), and in these patients adding cetuximab to FOLFIRI did not improve RR or PFS. In patients with tumor expressing wild-type KRAS, adding cetuximab to FOLFIRI improved median PFS (9.9 vs. 8.7 months for patients receiving FOLFIRI, p = 0.017), and RR (59.3% vs. 43.2% for patients receiving FOLFIRI alone, p = 0.0025). In contrast, there was no benefit at all in RR or PFS among patients with mutant K-RAS receiving FOLFIRI plus cetuximab vs. FOLFIRI alone.

The OPUS trial is a phase II study enrolling 337 patients and comparing FOLFOX (a regimen of 5-FU, LV and oxaliplatin) to FLOFOX plus cetuximab as first-line treatment in patients with metastatic CRC. The initial finding reported in 2007 ASCO annual meeting, showed an increased RR when cetuximab was added to FOLFOX, but this did not turn into better PFS [9]. In 2008 ASCO annual meeting, Dr. Carsten Bokemeyer presented the KRAS analysis of tumor tissues from 233 patients in this study, and KRAS mutation was detected in 42% [10]. In patients with wild-type KRAS tumor, RR was 61% in FOLFOX plus cetuximab group vs. 37% in FOLFOX (p = 0.011), and this turned into improvement in median PFS (7.7 months vs. 7.2 months, p = 0.016). In patients with mutant KRAS, RR was worse in FOLFOX plus cetuximab (33% vs. 49% in FOLFOX, p = 0.11), and this turned into significantly worse median PFS (5.5 months vs. 8.6 months in FOLFOX, p = 0.019).

Skin toxicity has previously been shown to correlate with clinical benefits such as RR, PFS and overall survival in patients with advanced CRC receiving anti-EGFR antibody [11]. The EVERST study is to determine whether dose-escalation of cetuximab based on skin toxicity in combination with irinotecan could improve efficacy in patients who failed irinotecan-based therapy. After 22 days of standard dose of cetuximab with irinotecan, patients with grade 0/1 skin reactions were randomized to receive combination of irinotecan plus either standard dose of cetuximab (250 mg/m^2 ^weekly), or escalated doses of cetuximab (50 mg/m^2 ^increase every 2 weeks till 500 mg/m^2 ^weekly or more than grade 2 skin toxicity). In 2007 ASCO annual meeting, Dr. Sabine Tejpar showed that increased dose of cetuximab improved RR, but was associated with a doubling of grade 3/4 diarrhea and grade 2 or higher skin toxicity [12]. In 2008 ASCO annual meeting, Dr. Tejpar presented a retrospective analysis of KRAS status in archived tumor tissues from 148 (including 77 of 89 randomized) patients in this study, and mutation was identified in 39% [13]. For patients with wild-type KRAS, the RR was 21.1% on standard cetuximab vs. 46.4% on escalated cetuximab doses. However, none of the patients with mutated KRAS in either arm achieved a response. The severity of skin rash did not have any association with KRAS status. The findings from this study suggest skin toxicity and KRAS status are independent predictors of outcome for anti-EGFR antibody treatment.

The retrospective analyses of KRAS data from CRYSTAL, OPUS and EVEREST have further demonstrated patients with K-RAS mutant CRC do not benefit from anti-EGFR antibody treatment. The addition of cetuximab to FOLFIRI or FOLFOX as first-line treatment only benefits patients with wild-type KRAS tumors, however the optimal sequence of biological therapy with chemotherapy in this population remains to be determined. The KRAS data has changed the paradigm of anti-EGFR antibody treatment in CRC. National Comprehensive Cancer Network has recently revised its CRC practice guideline, and recommends CRC patients with know KRAS mutations should not be treated with anti-EGFR antibody alone or in combination with other anticancer agents [14]. The European Medicines Agency has recognized these findings, and restricts the use of anti-EGFR antibody in CRC patients only with wild-type KRAS tumors.

National Cancer Institute (NCI) has suspended all ongoing U.S. cooperative group studies involving anti-EGFR antibody in CRC since June 2008 [15]. NCI has amended N0147 study, which is an adjuvant trial comparing cetuximab plus FOLFOX vs. FOLFOX in patients with stage III colon cancer after surgery. After amendment, only patients with wild-type KRAS tumors will be randomized for protocol treatment [16,17].

### Worse Outcome for Combined Anti-EGFR and Anti-VEGF Antibody Therapy in the First-Line Treatment

Fluoropyrimidine-based chemotherapy plus the anti-vascular endothelial growth factor (VEGF) monoclonal antibody bevacizumab is the standard front-line treatment for patients with advanced CRC. Data from the BOND2 study has demonstrated that the use of the bevacizumab and cetuximab in combination with irinotecan-based chemotherapy is feasible and potentially more efficacious than irinotecan plus cetuximab in patients with metastatic CRC refractory to irinotecan-based therapy [18]. The PACCE trial was conducted to examine the role of double antibody treatment in patients with advanced CRC by comparing bevacizumab and chemotherapy (FOLFOX or FOLFIRI) with or without panitumumab as initial treatment. The results of PACCE trial showed increased RR but inferior PFS in patients receiving double antibody with chemotherapy [19]. The interim analysis of KRAS status in the subgroup of FOLFIRI and bevacizumab has shown the increased RR associated with panitumumab was only seen in patients with wild-type KRAS.

In 2008 ASCO annual meeting, a second phase III randomized study, CAIRO-2, testing the role of combining EGFR and VEGF antibody with chemotherapy as the first -line treatment in patients with advanced CRC, was presented by Dr. Punt [20]. In this study, capecitabine, oxaliplatin, and bevacizumab with or without cetuximab were compared. Median PFS was significantly reduced in patients on double antibody with chemotherapy (9.6 months) compared with bevacizumab plus chemotherapy (10.7 months, p = 0.018), but there was no differences in RR and overall survival (OS) between these 2 groups. In patients with mutated KRAS, the addition of cetuximab to chemotherapy and bevacizumab resulted in significantly decreased PFS (8.6 months vs. 12.5 months, p = 0.043). There was no difference in PFS in those with K-RAS wild-type tumors.

Data from both PACCE and CAIRO-2 studies have indicated no benefit of adding anti-EGFR antibody to bevacizumab and chemotherapy in the first-line treatment of advanced CRC, and patients with mutant KRAS tumors had worse outcome on double antibodies and chemotherapy compared to bevacizumab and chemotherapy. As a result of these two reports, NCI has suspended two on-going phase III cooperative group studies, Cancer and Leukemia Group B (CALGB) 80405 and South West Oncology Group (SWOG) 0600, in June 2008 [16]. Both studies are designed to compare chemotherapy with double antibodies (EGFR and VEGF) or single antibody (EGFR or VEGF) in patients with metastatic CRC receiving first-line or second-line treatment. After a detailed analysis of toxicity date, CALGB 80405, which is a first-line study FOLFOX with bevacizumab, or cetuximab, or with the combination of bevacizumab and cetuximab in patients with metastatic CRC, has reactivated in December 2008. This study has reached approximately 60% of accrual goal (~2,300 patients). Combined biologic therapy with anti-EGFR and anti-VEGF antibodies is not recommended for patients with metastatic CRC outside of the clinical trial setting.

### Role of Pre-operative FDG-PET in Surgical Treatment of Colorectal Liver Metastases

Staging CRC patients by 2- [18F] fluoro-2-deoxy-D-glucose (FDG) and positron emission tomography (PET) is thought to be better than CT scan, however the evidence of improved clinical management and outcome is lacking. Wiering et al. reported a randomized controlled study enrolling 150 CRC patients with liver metastasis planning for surgical resection [21]. Patients were randomized to CT imaging only or CT and FDG-PET imaging before surgery for staging. Primary endpoint was futile laparotomy, defined as any laparotomy that revealed benign disease or that did not result in a disease free survival period longer than 6 months. Addition of PET to CT imaging identified 20% patients with benign or additional diseases before surgery and prevented 5 patients from surgery (2 benign diseases and 3 with extra-hepatic diseases). The number of futile laparotomy was reduced from 45% in the group without PET to 28% in the group with PET. This study concluded that addition of FDG- PET to the work-up for surgical resection of colorectal liver metastases prevented unnecessary surgery in one out of six patients. FDG-PET is recommended to be used routinely before planned liver resection for CRC metastases.

### FOLFIRI in Patients with Resected Liver Metastasis from CRC

Chemotherapy is frequently administered after complete resection of liver metastases from CRC, but the optimal regimen is yet to be established. Dr. Marc Ychou presented preliminary finding from CPT-GMA-301 study, which was conducted to compare 6-month of adjuvant chemotherapy with 5-FU/LV vs. FOLFIRI in this setting with DFS as the primary endpoint [22]. This randomized study enrolled 306 patients with complete resection of exclusively liver metastasis without prior treatment for metastatic disease. Prior adjuvant chemotherapy except irinotecan-based regimen was allowed. More grade-3/4 toxicities were observed in FOLFIRI arm, especially neutropenia. There was no statistical difference in 2-year DFS (46% for 5-FU/LV [95% confidence interval (CI), 38%–54%] vs. 51% with FOLFIRI [95% CI, 42%–58%] and 3-year OS (72% for 5-FU/LV [95% CI, 63%–79%] vs. 73% for FOLFIRI [95% CI, 64%–80%] between these 2 arms. However, there was a trend toward better outcome in FOLFIRI arm if patients started chemotherapy within 6 weeks of surgery (HR 0.75; p = 0.18). Therefore, randomized trial incorporating biological agents with chemotherapy is urgently needed in this setting to define the optimal regimen.

## Adjuvant chemotherapy in stage II and III colon cancer

### The Use of Bevacizumab

Allegra et al. reported the initial safety report of NSABP C-08, a phase III randomized study enrolling 2,710 patients to compare a modified FOLFOX regimen known as mFOLOX6 (every 2 weeks for 12 cycles) vs. bevacizumab and mFOLFOX6 (every 2 weeks for 12 cycles then bevacizumab alone every 2 weeks for 14 cycles) as adjuvant therapy in patients with stage II/III colon cancer after surgery [23]. A significantly higher percentage of patients completed 10 or more cycles of chemotherapy and received a higher cumulative oxaliplatin dose in the bevacizumab arm. The median duration of bevacizumab therapy was 11.5 months. Toxicities were well balanced in the 2 groups, with overall rate of grade 4/5 toxicities 15.2% and 15.3%, including death of 1.0% and 1.3%, respectively. There was no difference in treatment-associated mortality (excluding death after relapse or second primary) within 6 months or 18 months after randomization. Toxicities that were significantly increased in the bevacizumab arm included sensory neuropathy (which could be attributed by higher cumulative dose of oxaliplatin), hypertension, pain, proteinuria, hand-foot syndrome, and wound healing complications. There were no significant differences in the incidence of gastrointestinal perforation, hemorrhage, or arterial thrombotic events between these 2 arms. Long-term follow-up for efficacy and potential delayed side effects is ongoing.

### Role of Oxaliplatin

Wolmark and colleagues presented an update on the previously reported NSABP C-07 trial [24]. The trial was conducted to compared the efficacy of adjuvant chemotherapy with bolus 5-FU/LV vs. 5-FU/LV and oxaliplatin (FLOX) in patients with stage II and III colon cancer. Disease-free survival (DFS) was the primary endpoint and OS the secondary endpoint. The investigators previously reported a 3-year DFS that significantly favored FLOX over 5-FU/LV (76.5% and 71.6%, respectively, P = .004) at the 2005 ASCO annual meeting [25]. The 5-year OS was reported at the 2008 ASCO annual meeting. There was improvement of 5-year OS for FLOX (80.3%) vs. 5-FU/LV (78.3%), but not statistically significant (p = 0.061). Longer follow-up is needed to determine significant survival benefit in this study since patients with recurrent CRC are having longer survival due to the improvement of treatment outcome. This finding from NSABP C-07 is consistent with the results from the Multicenter International Study of FOLFOX in the Adjuvant Treatment of Colon Cancer (MOSAIC) reported in 2007 ASCO annual meeting [26]. In MOSAIC study, the OS in stage III patients was not statistically different till median of 6-year follow-up, with 4.4% better in FOLFOX (73.0%) vs. 5-FU/LV (68.6%; hazard ratio [HR] 0.80; p = 0.029). Both studies have confirmed the benefit of adding oxaliplatin to 5-FU based adjuvant chemotherapy for stage III colon cancer by showing superior 3-year DFS in oxaliplatin arm. Three-year DFS remains a very important endpoint and continues to be a surrogate endpoint for OS for adjuvant trial in colon cancer,

### Microsatellite Instability

The benefit of adjuvant chemotherapy is still debatable for stage II colon cancer. The United Kingdom QUASAR study has shown chemotherapy with 5-FU and LV in patients with stage II CRC can provide a small improvement (~4%) in rates of recurrence and OS compared to patients on observation [27]. However, in MOSAIC study, FOLFOX did not improve survival in patients with stage II colon cancer. This indicates a need for identifying high-risk patients with stage II colon cancer who may benefit from adjuvant chemotherapy. It has been shown that 5-FU-based adjuvant chemotherapy may not benefit patients with stage II or III colon cancer exhibiting microsatellite instability (MSI) [28].

In 2008 ASCO annual meeting, Sargent et al. presented an analysis of 491 patients from five clinical trials randomizing patients with stage II and III colon cancer to either 5-FU based adjuvant chemotherapy or no post-operative treatment [29]. Those with high MSI were stratified as having deficient mismatch repair (dMMR) and those with microsatellite stability or low MSI were stratified as having proficient mismatch repair (pMMR). Among these patients, stage II was 49% and dMMR was 15%. In patients with pMMR, adjuvant therapy with 5-FU translated into an increase in DFS and OS in stage III. Conversely, patients with dMMR derived no benefit from adjuvant 5-FU treatment either in stage II or III. This analysis was further pooled with the previously reported data by Ribic et al. [28] with patients number totaling 1,027, and the 5-year DFS and OS were worse in patients with stage II colon cancer with dMMR treated with 5-FU therapy vs. observation alone. This study has further supported that MSI can be used to predict who will benefit from adjuvant 5-FU chemotherapy for colon cancer, particularly in patients with stage II disease. The ongoing Eastern Cooperative Oncology Group (ECOG) 5202 study is a perspective study in stage II colon cancer to identify high-risk patients for adjuvant treatment using molecular marker analysis including MSI and tumor 18q loss of heterozygosity [30]. The result of ECOG 5202 will provide a definitive answer on how to use molecular markers in selecting high-risk patients for adjuvant treatment in patients with stage II colon cancer.

## Neurotoxicity and efficacy with intermittent oxaliplatin and use of calcium and magnesium

The sensory neuropathy associated with cumulative oxaliplatin treatment frequently interrupts with the administration of oxaliplatin-based regimen for CRC. Two studies presented at the 2008 ASCO annual meeting examined the strategy of using calcium and magnesium, and one study also examined intermittent administration of oxaliplatin in minimizing neurotoxicity from oxaliplatin.

The Combined Oxaliplatin Neurotoxicity Prevention Trial (CONcePT) randomized patients receiving first-line therapy for metastatic CRC to either continuous or intermittent FOLFOX plus bevacizumab. The intermittent arm differed from the continuous arm in that oxaliplatin was stopped after 8 cycles in patients who had at least stable disease, then was re-started after another 8 cycles of maintenance therapy with bevacizumab and infusional 5-FU/LV or tumor progression during maintenance treatment. Patients in both arms were also randomized to receive intravenous calcium gluconate and magnesium sulfate (CaMg) before and after oxaliplatin treatment. This study was discontinued prematurely due to interim analysis suggesting there were significantly lower RR in patients receiving CaMg. However, a subsequent independent radiology review did not find any evidence of detrimental effect from CaMg on the activity of FOLFOX plus bevacizumab. An analysis of the 139 patients who received treatment per protocol was performed and presented at 2008 ASCO annual meeting [31]. Time to treatment failure (TTF), the primary endpoint of the trial, was significantly longer in patients receiving the intermittent oxaliplatin schedule, 5.6 months vs. 4.2 months in those receiving continuous treatment (HR 0.58; p = 0.0025). Severe neurotoxicity was significantly reduced in the intermittent oxaliplatin arm (10%) compared with the continuous oxaliplatin arm (24%, p = 0.048). Treatment delays or dose reductions for neurotoxicity were more than twice as frequent in patients on the continuous oxaliplatin arm. There was no significant effect of CaMg or placebo on TTF or PFS. The investigators concluded that intermittent oxaliplatin administration was associated with a significant improvement of TTF compared with continuous oxaliplatin, without compromising PFS. CaMg reduced the severity of neuropathy and did not compromise the activity of FOLFOX plus bevacizumab. Taken together with data from the previously reported OPTIMOX1 & OPTIMOX2 trials [32,33], CONcePT trial provides further evidence that intermittent oxaliplatin-based therapy should be considered the standard of care in the first-line treatment of metastatic CRC.

N04C7 was designed as a placebo-controlled phase III study to prospectively evaluate the activity of CaMg as neuroprotectant against cumulative oxaliplatin-related peripheral sensory neurotoxicity [34]. Patients undergoing adjuvant FOLFOX chemotherapy were randomized to receive CaMg or placebo. This trial accrued only 104 of 300 planned patients due to the early closure of the CONcePT trial. Despite the early closure, a significantly decreased incidence of grade 2 or higher neurotoxicity was observed in patients receiving CaMg (22% vs. 41% in the placebo group, p = 0.038). The time to development of grade 2 or higher neurotoxicity was also prolonged in the CaMg group. Additionally, there was no difference in side effects between CaMg and placebo group. The finding from this study indicates that CaMg can be considered as a routine neuroprotective treatment when used in conjunction with oxaliplatin-based chemotherapy in the setting of adjuvant therapy for CRC.

## Non-colorectal gastrointestinal cancers

Non-colorectal gastrointestinal cancers have a significant burden world-wide. Modern multimodal approaches that integrate surgery, radiation and systemic therapy, the development of new cytotoxic agents along with anti-EGFR and anti-VEGF therapies have led to a significant survival improvement for colorectal cancer patients. Advances have been limited however, in the case of non-colorectal gastrointestinal cancers. The research presented at the 2008 ASCO annual meeting indicates that progress in these cancers is forthcoming. Abstracts from the annual meeting are grouped below into categories based on disease sites: pancreatic, esophagogastric, and hepatobiliary.

## Pancreatic cancer

### Adjuvant Therapy for Pancreatic Cancer

Surgical resection remains the only potential curative therapy for pancreatic cancer patients. However, 5-year survival for surgically resected patients is 30% only and most patients die of disseminated disease. Therefore, effective adjuvant strategies are needed. The Gastrointestinal Tumor Study Group (GITSG) 9173 trial indicated that post-operative 5-FU and radiotherapy extended the median overall survival to 20 months, as compared with 12 months with observation alone. Similar results were reported subsequently by the Johns Hopkins and European Organization for Research and Treatment of Cancer (EORTC) and 5-FU -based chemoradiation became the standard-of-care for nearly two decades [35]. The European Study Group for Pancreatic Cancer (ESPAC-1) has been however, a paradigm-changing trial which was a multinational effort conducted in 11 European nations [36]. This study indicated for the first time that adjuvant systemic chemotherapy led to a superior survival as compared with the either the no-chemotherapy or the chemoradiotherapy groups. Although the study methodology of ESPAC-1 is regarded as controversial, this trial has demonstrated that a median survival of 20 months could be achieved with adjuvant 5-FU chemotherapy alone, without the addition of radiation. Therefore, adjuvant chemotherapy is regarded as the standard adjuvant treatment after surgical resection of the pancreas in Europe. The recent Radiation Therapy Oncology Group (RTOG) 9704 indicated that adjuvant gemcitabine followed by chemoradiation was superior to 5-FU for pancreatic head carcinomas [37,38]. The CONKO-1 study was a multi-center, European trial which randomized 368 patients with surgically resected pancreatic cancer to post-operative gemcitabine for 6 months vs. observation. Previous analyses revealed the adjuvant gemcitabine to be well-tolerated and an improvement in DFS. The final analysis of the CONKO-1 study was presented at the 2008 ASCO annual meeting [39]. There was a statistically significant improvement in DFS (13 vs. 7 months) with adjuvant gemcitabine. The improvement in overall survival however, was very modest (2 month-improvement with gemcitabine). Five-year survival was 21% for gemcitabine and 0% with observation. Adjuvant gemcitabine chemotherapy effectively improved DFS, irrespective of lymph node, margin status or T stage. This study highlights: a. the improved survival of patients treated with surgery alone (20 months), b. the limited benefit provided by any adjuvant strategy and c. that adjuvant therapy is relatively ineffective in the prevention of early mortality (survival curves separate only after 18 months). The American College of Surgeons Oncology Group (ACOSOG) phase II study Z05031 explored a novel combination of 5-FU, cisplatin, interferon and radiotherapy in the adjuvant setting, based on the results of a previous study conducted by Picozzi, et al. which indicated an impressive OS with this regimen [40]. The ACOSOG Z05031 enrolled 90 patients, of whom only 56% received all 3 cycles of therapy due to treatment-related toxicity. Despite the use of an intensive chemoradiation strategy, local recurrences occurred in 46% of the patients. The median OS for all patients in this study was 27 months, which at first glance appears superior to historical standards. However, the relatively common grade 3 toxicities (96%) and modest survival improvement (4 months more than the treatment arm in CONKO-1) argues against the widespread use of this regimen.

Together, the CONKO-1 and the ESPAC-1 studies argue against the use of standard, post-operative, adjuvant radiotherapy for pancreatic cancer. Preoperative chemoradiation strategies that decrease margin-positive resections and pre-select patients with better cancer biology for resection deserve further exploration.

### Advanced Pancreatic Cancer

The addition of erlotinib to gemcitabine improved OS as compared with gemcitabine alone, in the PA.3 study, although the median survival increase was very modest (5.9 to 6.4 months with the addition of erlotinib) [41]. The addition of cetuximab or bevacizumab to gemcitabine, on the other hand did not result in any survival improvement. The AVITA study was a randomized, phase III study that included 607 patients with metastatic pancreatic cancer and explored the addition of bevacizumab to the gemcitabine + erlotinib combination[42]. Study participants received first-line treatment with gemcitabine, erlotinib and placebo or gemcitabine, erlotinib and bevacizumab.

There was no significant prolongation of survival with the addition of bevacizumab, although DFS was significantly improved (from 3.6 to 4.6 months). Bevacizumab was reported to be safe in this combination, despite an increase in the incidence of epistaxis, hypertension and proteinuria. Interestingly, there was no reported increase in thrombotic events with bevacizumab. The AVITA study suggests that antiangiogenic strategies may have merit in the treatment of advanced pancreatic cancer, although the margin of benefit with bevacizumab is modest. Therefore, it is imperative that we identify patient subgroups that may benefit from such an approach.

Kindler et al. investigated a multi-targeted strategy against both the EGFR and VEGF in a randomized phase II study. Pancreatic cancer patients (n = 139) received gemcitabine, bevacizumab and erlotinib or gemcitabine, bevacizumab and cetuximab [43]. They noted that early hypertension correlated with response. There was no significant difference between the two arms in either OS or PFS. Therefore, cetuximab or bevacizumab cannot be recommended for pancreatic cancer at the current time outside of an investigational setting.

Gemcitabine has become the standard therapy for advanced pancreatic cancer, since its approval almost a decade ago. Subsequent investigational strategies have included the addition of other, targeted agents to gemcitabine. There have been very few attempts to address the role of alternative cytotoxic agents (which may represent better platforms for the addition of targeted therapies) other than gemcitabine in the first-line setting. The FFCD group randomized 202 patients with advanced, untreated pancreatic cancer to gemcitabine or 5-FU plus cisplatin [44]. Patients received therapy until progression, after which they could cross to the opposite arm. There were no significant differences in survival between the two arms. One-year and two-year survival figures were also identical between the gemcitabine and 5-FU plus cisplatin arms. Although it is unlikely that 5-FU and cisplatin will replace gemcitabine due to toxicity concerns, these data provide the rationale for non-gemcitabine containing regimens in the first-line setting based on pharmacogenomic profile.

### Second-Line Therapy

There are no standard second-line regimens for advanced pancreatic cancer, after gemcitabine failure. However, capecitabine, capecitabine and oxaliplatin (CAPOX), and FOLFOX are commonly used. The CONKO-3 study randomized 168 patients who had gemcitabine-refractory pancreatic cancer to 5-FU, LV and oxaliplatin (OFF) or 5-FU and LV (FF) [45]. The study was powered at 90% to detect an improved OS by 2 months in the OFF arm. Both regimens were tolerable, with the exception of higher neuropathy in the OFF arm. The median OS in the OFF arm was 28 weeks, and that of the FF arm was 13 weeks, thereby fulfilling the study hypothesis. There was also a significant prolongation of PFS in the treatment arm (13 vs. 9 weeks). OFF should now be regarded as a standard second-line regimen for pancreatic cancer.

### Novel Agents for Pancreatic Cancer

Nanoparticle albumin-bound (Nab)-paclitaxel is a novel paclitaxel formulation which is currently approved for the treatment of breast cancer. Due to the nano-size and presence of albumin, which attracts the particle to tumor sites, the penetration of cytotoxic agents bound to the nanoparticle is very high, possibly leading to a better anti-tumor response. Preclinical data indicated that increased expression of Secreted Protein and Rich in Cysteine (SPARC) within tumors resulted in improved anti-tumor response to Nab-paclitaxel. Pancreatic cancer overexpresses SPARC protein and impressive partial responses (PRs) were seen in a phase I pancreatic cancer study of Nab-paclitaxel + gemcitabine [46]. Nine PRs occurred in 20 treated patients, after a median of 2 cycles. This combination appears to have promising activity in pancreatic cancer.

The CALGB presented the results of a single-arm phase II study of sunitinib for patients with advanced pancreatic cancer who had previously been treated with gemcitabine-based therapy. No responses were reported in 77 treated patients, and stable disease resulted in 7 patients [47]. The California consortium reported similar disappointing results with sorafenib when combined with gemcitabine [48]. In this randomized study, chemo-naïve pancreatic cancer patients received sorafenib as a single agent or as a combination with gemcitabine. No responses resulted with sorafenib alone and the median survival of the gemcitabine + sorafenib arm was 6 months only. Both sunitinib and sorafenib have insufficient anti-tumor activity in pancreatic cancer and do not merit further study in this disease. Mammalian Target of Rapamycin (mTOR) has emerged as an important target for therapy in renal cell carcinoma. Wolpin et al. treated 31 gemcitabine-refractory pancreatic cancer patients with the mTOR-directed oral agent, everolimus 10 mg once daily. Although the agent was tolerable, there were no responses and disease stability was uncommon [49].

### Locally Advanced Pancreatic Cancer

Gemcitabine in combination with radiotherapy (50.4 Gy in 28 fractions) was compared with gemcitabine chemotherapy (alone) for locally advanced pancreatic cancer by the Eastern Cooperative Oncology Group (ECOG) 4201 study. Although this study was closed early (74 patients enrolled out of planned size of 316 patients) due to poor enrollment, there was a statistically significant median-survival improvement in the combination-therapy arm (9.2 months with gemcitabine and 11 months with gemcitabine and radiation). Unfortunately, this very modest survival improvement occurred at the expense of 40% grade 4 gastrointestinal/hematological toxicities in patients receiving chemoradiation. Kim et al. reported the results of a phase II study of gemcitabine, oxaliplatin and radiotherapy, followed by gemcitabine chemotherapy in 53 patients with locally advanced pancreatic cancer [50]. The median survival reported in this trial was 9.3 months, at the cost of substantial toxicity. These studies argue against upfront chemoradiation for locally advanced pancreatic cancer. Chemoradiation may be best utilized after induction chemotherapy for locally advanced cancers. The GERCOR phase II and III trials have indicated that induction chemotherapy can identify patients who are most likely to benefit from radiotherapy [51]. Pancreatic cancer patients with stable disease after 3 months of chemotherapy received chemoradiation in these studies and their median survival was 15 months, as compared with 11 months for those receiving chemotherapy alone.

### Patient-Tailored Therapy for Pancreatic Cancer

Investigators at Johns Hopkins presented data on their preclinical model termed PanXenoBank, which uses surgically resected pancreatic adenocarcinoma to generate xenograft in mice [52].

This xenograft serves as an *in vivo *platform for drug testing and provides data that can be used prospectively to treat patients. Of the 90 patients whose cancers were xenografted, 18 failed to engraft and preliminary results in the rest indicated that a tailored-approach, using unconventional agents such as mitomycin-C was possible. While the data in this regard is premature and the technique is labor-intensive, this strategy holds promise. The MD Anderson group presented data on single nucleotide polymorphisms (SNPs) of genes involved in gemcitabine metabolism and showed that cytidine deaminase and deoxycytidine kinase alleles were associated with an improved survival, albeit at the cost of increased toxicity [53]. Collisson et al. investigated gene expression profiles from micro-dissected pancreatic resection specimens using Affymetrix GeneChips and identified prognostic groups, which were based on differentially expressed genes [54]. They concluded that the disparity of outcomes was due to intrinsic differences in tumor biology. These data open avenues for targeting specific subgroups with dose-intensive strategies.

## Esophagogastric cancers

### Preoperative vs. Post operative Adjuvant Therapy

The Japanese Oncology Group (JCOG) presented updated data from the JCOG 9907 study, which randomized 330 patients with stage II or III esophageal cancer to pre-or post-operative chemotherapy with 5-FU and cisplatin [55]. In the post-operative arm, 30% of patients could not complete chemotherapy, while 90% completed treatment in the pre-operative arm. Only 4/160 patients had complete pathologic response in the pre-operative arm. In an early analysis of the trial, there appeared to be a significant progression-free survival benefit in favor of the preoperative arm. In an updated analysis in November 2007, however there was no reported benefit in PFS. In a subgroup analysis, patients who were lymph node negative (N0), appeared to benefit from preoperative therapy, while the lymph node positive patients did not. In their prior study JCOG 9204, patients with lymph node metastases benefited from adjuvant chemotherapy [56]. These conflicting results along with the large number of cases who did not receive adjuvant therapy makes the findings of the present study hard to interpret. Thus, despite this relatively large study, it remains to be proven that pre-operative therapy is superior to post-operative adjuvant therapy for esophageal cancer.

### Preoperative Therapy for Esophageal Cancer-Choice of Chemotherapy

Irinotecan plus cisplatin, paclitaxel plus cisplatin, and 5-FU plus cisplatin are commonly used chemotherapeutic combinations along with radiotherapy for the preoperative treatment of esophageal cancer. The ECOG 1201 was a randomized phase II study for paclitaxel plus cisplatin vs. irinotecan plus cisplatin along with radiotherapy for these patients [57]. All patients had rigorous preoperative staging which included endoscopic ultrasound. There were no significant differences in survival between the two arms, irrespective of stage. Furthermore, the results did not appear to be superior to those with 5-FU plus cisplatin.

### Chemoradiation as Definitive Therapy for Squamous Cell Carcinoma of Esophagus

Stahl et al. reported the results of a randomized trial of chemoradiation ± surgery for operable squamous cell carcinoma of the esophagus [58]. In their earlier report in 2005, they reported no improvement in survival with the addition of surgery, although there was an improvement in local control. In their updated analysis, 5 and 10-year survival figures were presented [59]. There was no clear survival difference at both time points between the surgical and non-surgical arms. Response to induction chemotherapy was the most important predictor of survival. These long term follow-up data again suggest that esophagectomy with its morbidities cannot be routinely recommended for esophageal squamous cell cancer.

### Intraperitoneal Adjuvant Therapy for Gastric Cancer

Peritoneal relapse occurs frequently in surgically resected gastric cancer. Therefore, adjuvant intraperitoneal chemotherapy merits study. Kang et al. randomized 640 patients with gastric cancer that had involved the serosa (at intraoperative evaluation) to intraperitoneal cisplatin, early mitomycin-C (administered on day 1) after surgery and an extended schedule of doxifluridine (for 12 months) and cisplatin, vs. delayed mitomycin-C (3–6 weeks post-operative) and a 3 month course of doxifluridine [60]. The results indicated a significantly improved relapse free survival and OS in the intraperitoneal therapy arm. Unfortunately, this study had several experimental elements, including early administration of mitomycin-C, inclusion of cisplatin and prolonged doxifluridine administration besides the intraperitoneal therapy. Thus, it remains to be proven if the survival improvement in the experimental arm is secondary to intraperitoneal therapy only. A randomized study wherein intraperitoneal chemotherapy is the only experimental treatment is required to answer this question.

### Systemic Therapy for Advanced Gastric Cancer

Docetaxel, cisplatin and 5-FU (DCF) is a FDA-approved regimen for gastric cancer, based on the results of the V325 study, which proved the superiority of this regimen as compared with 5-FU and cisplatin [61]. However, the toxicity of this regimen limits its widespread use. Therefore, Ridwelski et al. investigated the activity of docetaxel and cisplatin administered on a 3 weekly schedule (without 5-FU) and compared the activity of this regimen with 5-FU and cisplatin in a phase III study [62]. A total of 273 patients were enrolled, and the primary study endpoint was prolongation of time to progression. At 12 months follow-up, the RR and PFS were virtually identical in the two arms and there was no significant difference in OS. Neutropenia occurred more frequently with docetaxel and cisplatin, while diarrhea was more likely with 5-FU and cisplatin. DCF remains an acceptable standard regimen based on these results. Alteration to a weekly schedule (from 3-weekly) may ameliorate the toxicities of DCF.

### Novel Agents for Esophagogastric Cancers

Cetuximab was investigated in second-line setting as a single-agent in patients with metastatic esophageal cancer by SWOG [63]. Marginal activity was noted with a PFS of less than 2 months and OS of 4 months. When combined with systemic chemotherapy however, in a randomized phase II study of cetuximab ± cisplatin and 5-FU for metastatic squamous cancers of the esophagus, there was an improved survival noted in the cetuximab containing arm (9.5 vs. 5.5 months) [64]. S1 is an active agent in gastric cancer and has synergistic anti-tumor activity with cisplatin and docetaxel (table 1).

**Table 1:** S1 Studies in Advanced Gastric Cancer

**Abstract/author**	**Study agents**	**Patient number**	**Study Design**	**RR**	**PFS**	**OS**
A4534/ Jeung et al.	S1, Docetaxel, Cisplatin	80	Randomized phase II Docetaxel + S1 or Docetaxel + cisplatin	44% 24%	198 days 143 days (p = 0.03)	NR

A4533/ Jin et al.	S1 Cisplatin 5-FU	230	Randomized phase III S1 vs. S1 + cisplatin vs. 5-FU + cisplatin	25% 38% 19% (p = 0.021)	267 days 433 days 309 days (p = 0.008)	NR

A4537/ Sato et al.	S1 Docetaxel Cisplatin	31	Phase II study	87%	7.7 months	19 months

RR: Response rate; PFS: Progression-free survival; OS: Overall survival; NR: Not reported.

## Hepatobiliary cancers

### Sorafenib

Sorafenib is a novel inhibitor of Raf kinase, VEGF receptor (VEGFR) and platelet-derived growth factor receptor, and has been recently approved for the treatment of advanced hepatocellular cancer (HCC), based on the results of the SHARP trial [65]. In this study, patients with child's A cirrhosis were enrolled and an approximate 3-month survival benefit was demonstrated in the sorafenib group vs. the placebo group. In 2008 ASCO annual meeting, the effectiveness of sorafenib was investigated in subpopulations of hepatocellular carcinoma.

Raoul et al. studied the effect of sorafenib in ECOG performance status (PS) 1 and 2 patients as a subanalysis from the SHARP trial [66]. They noted that sorafenib was tolerable in this group of patients. However, it remains unclear if this advantage is applicable to ECOG PS 2 cases specifically as this group of patients are often candidates for supportive care only.

In Asian countries, the incidence of HCC is higher than in the western nations and is more likely to be HBV-associated. Cheng et al. randomized 226 Asian patients with HCC to sorafenib or placebo. Sorafenib significantly prolonged OS and PFS as compared with placebo [67]. The degree of benefit seemed to be similar to that experienced by the western patients, thus establishing this agent as a standard therapy for HCC.

Abou-Alfa et al. presented the results of sorafenib therapy in HCC patients with child's B cirrhosis [68]. In a phase II study, 137 HCC patients with child's A/B cirrhosis received the standard dose of 400 mg bid. They noted comparable sorafenib pharmacokinetic profiles of child's A and B groups. The median OS for child's B cases was 14 weeks and time to progression (TTP) was 13 weeks. There was a more frequent worsening of hepatic function in the child's B cases. The utility of sorafenib in this group of HCC patients remains to be proven.

### Sunitinib

Sunitinib is a multi-targeted tyrosine kinase inhibitor that is directed against VEGFR2 and has activity comparable to that of sorafenib. Zhu et al. presented phase II data of 34 patients with HCC who received sunitinib in a dose of 37.5 mg daily. Median PFS was 4 months and OS 10 months [69]. Biomarkers may correlate with response; elevated VEGF-C levels were associated with an improved TTP and OS. Treatment with sunitinib decreased tumor vascular permeability and plasma VEGFR2 levels. Sunitinib is an active agent in hepatocellular carcinoma, and may represent an alterative to sorafenib for advanced disease. A phase III randomized study comparing sunitinib vs. sorafenib as the first-line systemic treatment in patients with inoperable HCC is currently underway.

### Cholangiocarcinoma/periampullary cancer

Bevacizumab and erlotinib may have promising activity in this chemo-refractory disease. In a preliminary analysis of their multi-center phase II study, Holen et al. reported 4 PRs resulting from this biologic-only combination among 20 assessed patients, and 4 cases with stable disease [70]. Cetuximab in combination with gemcitabine and oxaliplatin resulted in either disease response of stability in the majority of cholangiocarcinoma patients; 6/22 patients who were initially considered unresectable had surgical resections after major responses [71]. Sorafenib results in disease stabilization in 30% of cholangiocarcinoma patients in a phase II study reported by Dealis et al. [72]. Toxicities however, were common in patients with ECOG PS 2. Cholangiocarcinoma and gall bladder cancers appear to be responsive to EGFR and VEGF inhibitors. A randomized study is required to determine the role of targeted agents in this disease.

Periampullary cancers may have a better prognosis as compared with pancreatic and biliary cancers. However, the standard of care in this patient population remains to be defined, due to the relative rarity of this disease. Overman et al. reported the efficacy results of a phase II study of CAPOX in advanced periampullary cancer. An impressive 50% RR and a median survival of 20 months was recorded [73]. Grade 3/4 toxicities were fatigue, neuropathy and myelosuppression. The addition of EGFR or VEGF targeted therapy to CAPOX is worthy of study in this disease.

## Competing interests

The authors declare that they have no competing interests.

## Authors' contributions

Both authors participated in drafting and editing the manuscript. Both authors read and approved the final manuscript.
